# Research on the Forward and Reverse Calculation Based on the Adaptive Zero-Velocity Interval Adjustment for the Foot-Mounted Inertial Pedestrian-Positioning System

**DOI:** 10.3390/s18051642

**Published:** 2018-05-21

**Authors:** Qiuying Wang, Zheng Guo, Zhiguo Sun, Xufei Cui, Kaiyue Liu

**Affiliations:** College of Information and Communication Engineering, Harbin Engineering University, Harbin 150001, China; wangqiuying@hrbeu.edu.cn (Q.W.); guozhengfield@hrbeu.edu.cn (Z.G.); xufeicui@hrbeu.edu.cn (X.C.); liukaiyue111@hrbeu.edu.cn (K.L.)

**Keywords:** inertial sensor, pedestrian position, adaptive zero-velocity interval adjustment, forward and reverse calculation

## Abstract

Pedestrian-positioning technology based on the foot-mounted micro inertial measurement unit (MIMU) plays an important role in the field of indoor navigation and has received extensive attention in recent years. However, the positioning accuracy of the inertial-based pedestrian-positioning method is rapidly reduced because of the relatively low measurement accuracy of the measurement sensor. The zero-velocity update (ZUPT) is an error correction method which was proposed to solve the cumulative error because, on a regular basis, the foot is stationary during the ordinary gait; this is intended to reduce the position error growth of the system. However, the traditional ZUPT has poor performance because the time of foot touchdown is short when the pedestrians move faster, which decreases the positioning accuracy. Considering these problems, a forward and reverse calculation method based on the adaptive zero-velocity interval adjustment for the foot-mounted MIMU location method is proposed in this paper. To solve the inaccuracy of the zero-velocity interval detector during fast pedestrian movement where the contact time of the foot on the ground is short, an adaptive zero-velocity interval detection algorithm based on fuzzy logic reasoning is presented in this paper. In addition, to improve the effectiveness of the ZUPT algorithm, forward and reverse multiple solutions are presented. Finally, with the basic principles and derivation process of this method, the MTi-G710 produced by the XSENS company is used to complete the test. The experimental results verify the correctness and applicability of the proposed method.

## 1. Introduction

Pedestrian-positioning technology based on the foot-mounted micro inertial measurement unit (MIMU) plays an important role in the field of indoor navigation [1,2,3,4,5,6]. MIMUs, which include gyroscopes and accelerometers, are installed on one foot of a pedestrian. MIMUs are used to measure the angular velocity and linear acceleration information in real time, and the positioning information is calculated using the dead reckoning method [7,8]. This pedestrian-positioning technology is passive, autonomous, not susceptible to outside interference, and suitable for the indoor environment without a global navigation satellite system for applications such as firefighting and military action.

However, the measurement accuracy of MIMUs is low because of the system noise [9], which causes the device measurement error to cumulatively diverge during dead reckoning. As a result, the pedestrian-positioning accuracy is significantly reduced. To restrain the rapidly diverging positioning error, a ZUPT method is generally proposed and widely used [10,11]. Its principle is that, on a regular basis, the foot is stationary in the ordinary gait; this is intended to reduce the position error growth of the system [12]. The pedestrian’s gait is detected from the output of the accelerometers and gyroscopes to determine whether the foot is moving. If the foot is moving, the dead reckoning algorithm is used to solve the positioning information. If the foot is stationary, the Kalman filter, which uses the velocity as an observation, is used to estimate the positioning error and compensate the location information. Therefore, the positioning error caused by the integrated measurement error can be limited.

Nevertheless, the traditional ZUPT has poor performance because the time of foot touchdown is short when the pedestrians move faster, which results in low positioning accuracy. Actually, ZUPTs consist of zero-velocity detection and error correction and both of them will be significantly affected by the velocity of pedestrian movement.

The zero-velocity detection is used to determine whether the pedestrian’s foot is touched down or lifting. Many scholars have conducted relevant research work, e.g., the magnitude detector and generalized likelihood ratio test detector [13,14,15,16]. However, regardless of the detector, the premise of ZUPTs is the use of a determined interval of the detected data (angular velocity and acceleration). In other words, it is important for the accuracy of the zero-velocity detection to determine the number of times N of foot motion. Particularly, there is big difference in the length of the touchdown time when the pedestrian is walking and running; then, the method to select the N value according to different states of motion is an important problem for zero-velocity detection.

The error correction is based on the following principle: on a regular basis, the foot is stationary during the ordinary gait to reduce the positioning error growth of the system. In theory, a longer error correction time has better convergence of the optimal estimation, better error compensation effect and higher positioning accuracy [17,18,19]. However, the length of the error correction time is directly affected by the velocity of pedestrian movement. In other words, a short interval of ZUPT when a pedestrian is walking or running will result in a short error-estimating time. Furthermore, the short interval of the ZUPT restrains the convergence of the optimal estimation algorithm and reduces the positioning accuracy of pedestrians. Therefore, completing the high-accuracy pedestrian-positioning error correction is a difficult problem for ZUPTs when the error correction time is short. To solve this problem, in [20], a smoothing algorithm for ZUPT-aided inertial navigation system was proposed. For near-real-time applications, smoothing is applied to the data in a step-wise manner, which requires a suggested varying-lag segmentation rule. Although this article proposes a reverse calculation method, the time to reverse the estimation only occurs once, whereas the smoothing part of this method is only filtering. There is no further discussion on its suitability for any velocity. In other words, it was implemented and tested only for slow walking. Therefore, it is necessary to further examine the method to adaptively determine the smoothing number in different moving states.

Considering these problems, a forward and reverse calculation method based on adaptive zero-velocity interval adjustment for the foot-mounted MIMU location method is proposed in this paper. This method aims at adaptively adjusting the algorithm of the ZUPT under different pedestrian velocities to improve the positioning accuracy of the navigation system. The paper is organized as follows: Section 2 introduces the basic principle of the pedestrian-positioning method based on the foot-mounted MIMU, including dead reckoning and ZUPT, and the effect of different moving velocities on the pedestrian-positioning accuracy is analyzed. Section 3 proposes a forward and reverse calculation based on the adaptive zero-velocity interval adjustment for the foot-mounted MIMU location method, which includes an adaptive adjustment for the zero-velocity interval method and forward and reverse calculation for the ZUPT method to adaptively adjust the length of the zero-velocity interval and optimal estimation for the positioning error under the condition of high-speed movement. Section 4 uses the MTi-710 device as the test sensor, and the proposed calculation method is verified in principle. Section 5 summarizes the conclusions of the study.

## 2. Basic Principles of Pedestrian Positioning Based on the Foot-Mounted MIMU

### 2.1. Gait Classification

**Figure 1:**
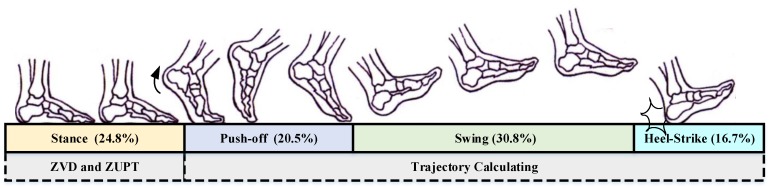
Four phases of a gait cycle. ZVD: zero-velocity detector; ZUPT: zero-velocity update.

The periodic action of pedestrian walking is called gait, and each gait cycle can be divided into four phases: stance phase, push-off phase, swing phase, and heel-strike phase [20,21], as illustrated in Figure 1. The percentage of time involved in the stance phase, push-off phase, swing phase, and heel-strike phase is approximately 24.8%, 20.5%, 38.0%, and 16.7%, respectively [22,23].

- The stance phase represents the period of time during which the foot is flat on the ground, and the weight of the body is directly over the supporting limb;
- The push-off phase is where only the big toe of the forward or reference limb is in contact with the ground;
- The swing phase indicates the period of time when the foot is no longer in contact with the ground, and the limb is free to move;
- During the heel-strike phase, the heel of the forward or reference foot touches the ground.

There are statistics of gait characteristics of ordinary people in different states of motion as illustrated in Table 1 [24,25,26]. The sampling frequency of the foot-mounted MIMU is 100 Hz, and the length of the data is the product of the sampling frequency and the stance phase time.

### 2.2. Basic Principles of Pedestrian Positioning

#### 2.2.1. Dead Reckoning and Error Correction

**Figure 2:**
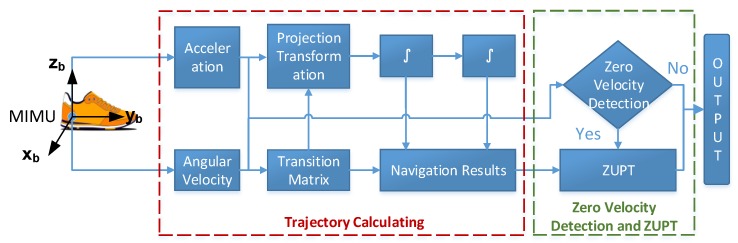
Basic principle of the dead reckoning algorithm based on ZUPT.

The pedestrian-positioning method based on the foot-mounted MIMU installs the sensor on the foot of pedestrians, so the trajectory information is obtained through the acceleration and angular velocity measured by the MIMU during the foot movement. Furthermore, the zero-velocity detector (ZVD) and ZUPT method are used to correct the calculation error of the positioning system to improve the positioning accuracy. The basic principle of the dead reckoning algorithm based on the ZUPT is shown in Figure 2.

According to the basic positioning principle illustrated in Figure 2, the calculation process of pedestrian location based on the MIMU consists of two parts: dead reckoning and error correction.

- Dead reckoning: the dead reckoning method obtains the trajectory from the acceleration and angular velocity information of the pedestrian’s foot, which is measured by MIMUs. However, the relatively low accuracy of the measurement is one of the shortcomings of MIMUs, particularly in the integration process of track calculation (red box in Figure 2). The device measurement error is accumulated and enlarged, which significantly reduces the pedestrian tracking results.
- Error correction: to suppress the error in track calculating, the error correction method, which includes the ZVD and ZUPT (green frame in Figure 2) algorithm, is introduced. This method, i.e., the optimal estimation method, is used to correct the accumulated error of the inertial sensor caused by the measurement error during the integration. The optimal estimation and compensation for the positioning error is processed through the ZUPT with the ZVD to determine whether the foot is landing. The basic principles and existing problems of the ZVD and ZUPTs are explained in detail in the next two parts.

#### 2.2.2. ZVD and ZUPT

(1) Basic Principle of the ZVD

ZVD is a detection method based on the foot motion state in pedestrian movement, where the amplitude detector is the most basic method of zero-velocity detection. Theoretically, when the pedestrian is in the zero-velocity state, where the foot is in the stance phase, the amplitude of acceleration measured by the MIMU is equal to the amplitude of the local gravity acceleration, and the angular velocity value is zero. The core formula of the ZVD with this principle is as follows:

(1) $$T(Z_{k}^{a})=\frac{1}{\sigma_{a}^{2}W}\sum_{l=k}^{k-W+1}\left(∥f(l\right)∥-g{)}^{2}$$

(2) $$T(Z_{k}^{w})=\frac{1}{\sigma_{\omega }^{2}W}\sum_{l=k}^{k+W-1}∥\omega (l){∥}^{2}$$

where f(l) and ω(l) are the measurement values of the accelerometer and gyroscope, respectively; ∥ ∥ denotes the modulo operator; g is the local gravity acceleration; $\sigma_{a}^{2}$ and $\sigma_{\omega }^{2}$ are the variance of the measurement noise of the accelerometers and gyroscopes, respectively; and W is the window size of the ZVD.

Because of the measurement noise, the results of Formulas (1) and (2) cannot be directly compared with zero but are compared with the pre-set threshold: when the calculation result is less than the threshold, the zero-velocity state is determined; and when the calculation result is greater than the threshold, the non-zero-velocity state is determined. The threshold value is obtained by processing the data in the static state and set based on many tests.

To improve the accuracy of the ZVD, the generalized likelihood ratio test detector and zero-velocity detection method based on fuzzy logic are proposed in [12,13,14,15] based on the magnitude detector. However, regardless of how the ZVD method is improved, the window size parameter does not change. In other words, a determined interval length of the data for the MIMU is the precondition of ZVD. When W increases, the detection accuracy can be improved because of the fullness of the measured data, but the real-time performance of the system is reduced; in contrast, if W is small, the detection accuracy is reduced, whereas the real-time performance of the system is improved. Therefore, W must be adjusted according to the actual situation.

(2) Basic Principle of ZUPT

When the pedestrian gait is detected as stationary, the velocity of the foot should be theoretically zero, i.e., the velocity calculated by the inertial navigation system should be zero. However, the actual output of the positioning system is not zero because of the device error and algorithm error. The velocity value obtained by the dead reckoning algorithm is essentially the velocity error of the pedestrian, so the Kalman filter can be used to correct the navigation error using the velocity error as the observation [27]. Based on this principle, the system state equation and measurement equation are established as follows:

(3) $$\begin{array}{l} \dot{X}=AX+\eta_{k}\\ Z=HX+\nu_{k} \end{array}$$

where $X={\left[\begin{array}{ccc} \delta p^{T} & \delta v^{T} & \delta \varepsilon^{T} \end{array}\right]}^{T}$ is the state vector in the Kalman filter; δp, δv and δε are the position error, velocity error and attitude error, respectively. A is the system transfer matrix, whose form is described in paper [2]; $H=\left[\begin{array}{ccc} 0_{3\times 3} & I_{3\times 3} & 0_{3\times 3} \end{array}\right]$ is the observing matrix; Z is the observed vector in the Kalman filter; and $\eta_{k}$ and $\nu_{k}$ are the process noise and observation noise, respectively.

Then, the velocity error is used as the observation based on the establishment of the system state equation and measurement equation, and Kalman filtering is used to perform the optimal estimation and compensation for each navigation error state quantity to improve the pedestrian-positioning accuracy. However, the more adequate filtering time corresponds to the more accurate state quantity estimate according to the basic principle of Kalman filtering. In other words, the covariance matrix does not converge when the filtering time is too short, which will significantly reduce the filtering accuracy. Therefore, we must further examine whether the touchdown time satisfies the requirement of the Kalman filter because of the limited landing time of a pedestrian foot.

According to the description and division of the pedestrian gait, the pedestrian foot touchdown interval that the ZVD and ZUPT process is called the stance phase, which accounts for approximately 24.8% of the walking pace. Table 1 shows that when the pedestrians accelerate, the duration of one gait cycle shortens, the stance phase time also shortens, and the length of data that can be used for the zero-velocity detection and zero-velocity correction shortens. Particularly for the running phase, the data length is shorter; e.g., when the moving speed is approximately 15 km/h, the total data length for ZVD and ZUPT is only 5. For such a short data length, the method to conduct the ZVD and ZUPT to ensure the accuracy of pedestrian positioning requires further study.

## 3. Zero-Velocity Detector Based on Adaptive Detection Interval

### 3.1. Performance Analysis of the Gait Feature

The acceleration and angular velocity are the core physical quantities measured in real time during pedestrian positioning: this information is used to determine the state of the foot and calculate the pedestrian-positioning information. On this basis, this paper introduces two parameters, jerk and angular acceleration. In engineering, jerk is used to characterize the speed of force changes and the angular acceleration is used to characterize the speed of the angular velocity changes. Jerk and angular acceleration are calculated as follows:

(4) $$\dot{f}{}^{b}\left(k\right)=\frac{\sqrt{{\left\|f^{b}\left(k\right)-f^{b}\left(k-1\right)\right\|}^{2}}}{T}$$

(5) $$\dot{\omega }{}^{b}\left(k\right)=\frac{\sqrt{{\left\|\omega^{b}\left(k\right)-\omega^{b}\left(k-1\right)\right\|}^{2}}}{T}$$

where $\dot{f}{}^{b}\left(k\right)$ is the jerk at time *k*, which describes the rate of acceleration changing; $\dot{\omega }{}^{b}\left(k\right)$ is the angular acceleration at time *k*; the marker *b* denotes the projection along the foot coordinate system; and T is the sampling time.

**Figure 3:**
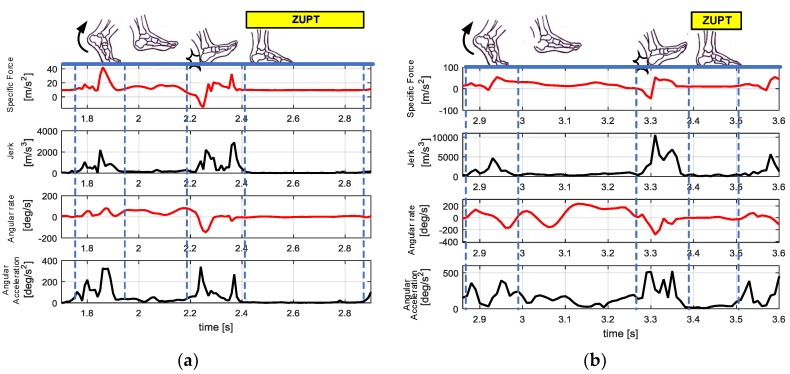
Variations of the foot motion parameters during one gait cycle in different states. (**a**) Walking; (**b**) jogging.

The acceleration and jerk along the $z_{b}$ axis and the angular velocity and angular acceleration along the $y_{b}$ axis in one gait cycle under walking or jogging conditions are shown in Figure 3. The $z_{b}$ axis acceleration and $y_{b}$ angular velocity are selected because the parameters of these two axes most obviously change with the change in gait.

Here, specific force, the measurement obtained from IMU, will be transformed into measurement of acceleration by accommodating for the gravity.

Figure 3 shows that walking and running have similar magnitudes of acceleration (within 100 m/s^2^) and similar orders of magnitude for the angular velocity and angular acceleration. However, the maximum jerk magnitude is close to 10^4^ when the pedestrian is running but approximately 10^3^ during walking. Therefore, the pedestrian can be determined to be walking or running by calculating the jerk and maximum jerk of the foot in real time. Then, we will discuss how to use the jerk to accurately determine the pedestrian movement status in detail.

### 3.2. Adaptive Adjustment for the Zero-Velocity Interval Method

**Figure 4:**
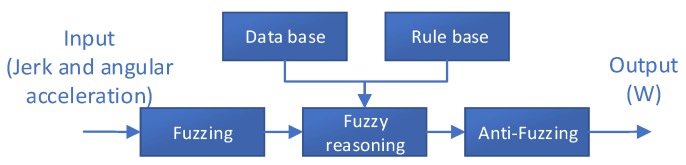
Basic logic diagram of fuzzy logic control.

To fully use the information of jerk and angular acceleration, this paper uses a fuzzy logic-based judgment method to accurately determine the pedestrian movement status. Fuzzy logic is a type of conceptual judgment and reasoning mode of thinking that imitates the human brain. For a system with unknown or uncertain model, the fuzzy set and fuzzy rules are used for reasoning, and transition boundaries or qualitative knowledge experiences are expressed. Fuzzy logic can be used to implement fuzzy comprehensive judgments. The basic block diagram of fuzzy logic control is shown in Figure 4.

(1) Fuzzy Processing of Data

Fuzzy processing is used to standardize the input, i.e., the input variable range is mapped to the corresponding domain; then the input data in the domain are transformed into the corresponding linguistic variables to form a fuzzy set so that the input value is converted into a fuzzy input variable, which is defined by a membership function. In this paper, the jerk is used as the input quantity and discretely defined as several levels in the following form:

$$\begin{array}{cc} U & =\{\mathrm{Low}\;\mathrm{Speed}\;\mathrm{Walking}\;\mathrm{High}\;\mathrm{Speed}\;\mathrm{Walking}\;\mathrm{Low}\;\mathrm{Speed}\;\mathrm{Running}\;\mathrm{High}\;\mathrm{Speed}\;\mathrm{Running}\}\\ & =\{\mathrm{LW}\;\mathrm{HW}\;\mathrm{LR}\;\mathrm{HR}\} \end{array}$$

(2) Knowledge Base

**Figure 5:**
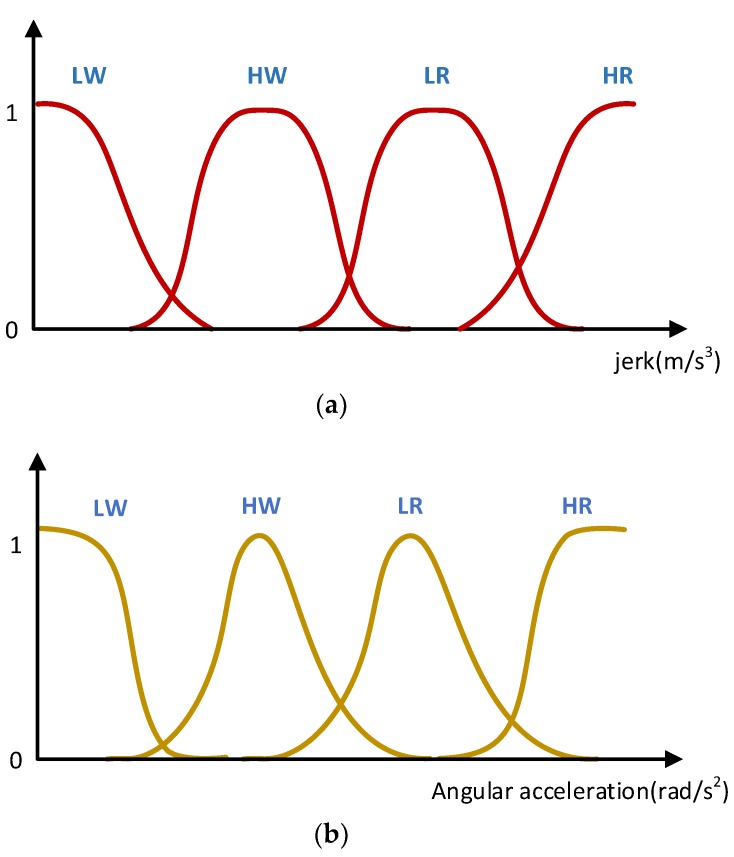
Two membership functions: (**a**) jerk membership function; (**b**) angular acceleration membership function.

The knowledge base includes the database and fuzzy library rules. The database stores the membership function values of all fuzzy subsets of the input and output variables and is responsible for providing data to the fuzzy inference engine in the fuzzy relational operation of fuzzy rule inference. The fuzzy rule base is used to store fuzzy control rules and provides the fuzzy inference engine with control rules during fuzzy inference. In this paper, the jerk membership function and angular acceleration membership function are shown in Figure 5.

For the jerk, the Z function is introduced as its membership function. For the angular acceleration, the ∏ function is introduced as its membership function. The three necessary fuzzy functions are shown as follows:

1. Z function
  (6) $$G_{F}\left(F_{i}\right)=\exp\left(\frac{-{\left(F_{i}-m_{i}\right)}^{2}}{2\sigma_{i}^{2}}\right)$$
2. S function
  (7) $$S_{F}\left(F_{i};a,b\right)=\{\begin{array}{cc} 0 & F_{i}<a\\ 2{\left(\frac{F_{i}-a}{b-a}\right)}^{2} & a\leq F_{i}<\frac{a+b}{2}\\ 1-2{\left(\frac{F_{i}-a}{b-a}\right)}^{2} & \frac{a+b}{2}\leq F_{i}<b\\ 1 & F_{i}\geq b \end{array}$$
3. ∏ function
  (8) $$\rho_{F}\left(F_{i};a,b\right)=\{\begin{array}{cc} S_{F}\left(F_{i};b-a,b\right) & F_{i}<b\\ 1-S_{F}\left(F_{i};b,b+a\right) & F_{i}>b \end{array}$$

where $F_{i}$ represents a fuzzy subset; $m_{i}$ denotes the mean for Gaussian distribution; $\sigma_{i}$ represents the expectation for Gaussian distribution; a, b are the relevant parameters of the fuzzy inference system and the specific value need to be set according to a large number of experimental results.

Having determined the appropriate membership function, fuzzy inference is used to detect the pedestrian’s movement state.

(3) Fuzzy Inference Engine

The fuzzy inference engine is the core of the fuzzy controller. It imitates the characteristics of human thinking; according to the fuzzy control law, which has been prepared in advance by expert knowledge and experience, fuzzy mathematics theory is used to calculate and infer the fuzzy control law to obtain a qualitative quantity expressed in words. In this paper, by using fuzzy inference methods and building a knowledge base of membership functions, we infer that the current state is slow walking, fast walking, jogging, or running based on the input information of the jerk. We define this state by the symbol U.

(4) Defuzzification Processing

According to the results obtained by the fuzzy inference engine, the size of the corresponding ZVD sliding window can be selected. The selection method is as follows:

(9) $$W=\{\begin{array}{lll} 2, & \mathrm{if} & U=\mathrm{HR}\\ 3, & \mathrm{if} & U=\mathrm{LR}\\ 4, & \mathrm{if} & U=\mathrm{HW}\\ 5, & \mathrm{if} & U=\mathrm{LW} \end{array}$$

**Figure 6:**
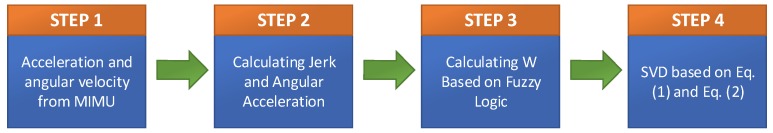
Basic implementation steps of the ZVD based on the adaptive detection interval.

Finally, the fuzzy logic control method is used to obtain the adaptively adjusted window size of the ZVD based on the angular velocity and acceleration information of the pedestrian foot, which is measured by the MIMU as the data source. The basic implementation steps of the ZVD based on the adaptive detection interval is shown in Figure 6.

## 4. Forward and Reverse Multiple Solutions for ZUPT

**Figure 7:**
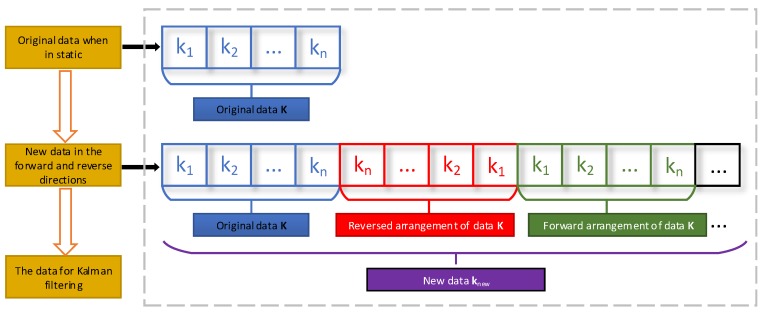
Basic block diagram of the ZUPT based on forward and reverse multiple solutions.

The Kalman filter is used to correct the error when a pedestrian is in the zero-velocity status, which is determined by the adaptive ZVD method. The velocity error is introduced as an observation to optimally estimate and correct the positioning error. However, according to Table 1, the foot zero-velocity time is short when the pedestrian moves fast, the error correction data is scant, and the estimation of each state variable based on the filter cannot converge. To solve this problem, zero-velocity based on forward and reverse multiple solutions is proposed in this paper. The basic block diagram of the ZUPT based on forward and reverse multiple solutions is illustrated in Figure 7.

First, the data interval of pedestrian zero velocity is determined by the ZVD; i.e., we obtain the data k_1_, k_2_, …, k_n_ in Figure 7 and name the data K; at time n, we process the data as shown in Figure 7, reversed arrange the data K in chronological order, named data K1 (k_n_,…, k_2_, k_1_,); then, we alternately arrange data K and data K1 to form new data $K_{\mathrm{new}}$, which are introduced as the MIMU measurements on the foot zero-velocity status. According to the method in Section 2.2, the new data are used to correct the positioning error based on dead reckoning. The data can be reorganized because the velocity of the foot is zero at this time. However, the forward and reverse directions are used here to ensure the continuity of the measurement noise. The specific calculation process is shown as follows:

By rearranging the data in the zero-velocity interval, a long set of data $K_{\mathrm{new}}$ is obtained, which is s times longer than the original data.

(10) $$K_{\mathrm{new}}=\left[\underset{\mathrm{sn}}{\underset{︸}{\begin{array}{ccccc} K & K1 & K & K1 & \cdots \end{array}}}\right]$$

where n denotes the length of the zero-velocity interval, and s represents the number of times that data is repeated in forward and reverse directions.

After that, Kalman filter is performed s times in succession. The first filtering result is used as the initial information of the second filter, the second filtering result is used as the initial information of the third filter, and so on, until the final result is obtained. In each process of Kalman filtering, the three-axis position error, three-axis velocity error, and three-axis attitude error of pedestrian $X={\left[\begin{array}{ccccccccc} \delta p_{x} & \delta p_{y} & \delta p_{z} & \delta \nu_{x} & \delta \nu_{y} & \delta \nu_{z} & \delta \varepsilon_{x} & \delta \varepsilon_{y} & \delta \varepsilon_{z} \end{array}\right]}^{T}$ are adopted as state quantities. The velocity error $Z={\left[\begin{array}{ccc} \delta \nu_{x} & \delta \nu_{y} & \delta \nu_{z} \end{array}\right]}^{T}$ is adopted as observation quantities.

The forward and reverse calculation method is used to overcome the defect that the low position accuracy for normal ZUPT is due to the incomplete convergence of the filter parameters. This has the advantages of simple principle and easy implementation. Experiments shows that this method is correct and effective and can obtain ideal positioning results.

## 5. Performance Evaluation

To verify the correctness and effectiveness of the forward and reverse calculation method based on the proposed adaptive zero-velocity interval adjustment in this paper, an MTi-G710 MIMU produced by XSENS company in the Netherlands is used.

**Figure 8:**
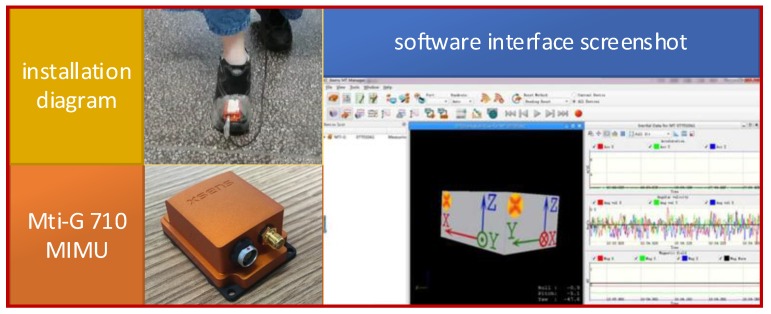
Device diagram for pedestrian-positioning test.

**Figure 9:**
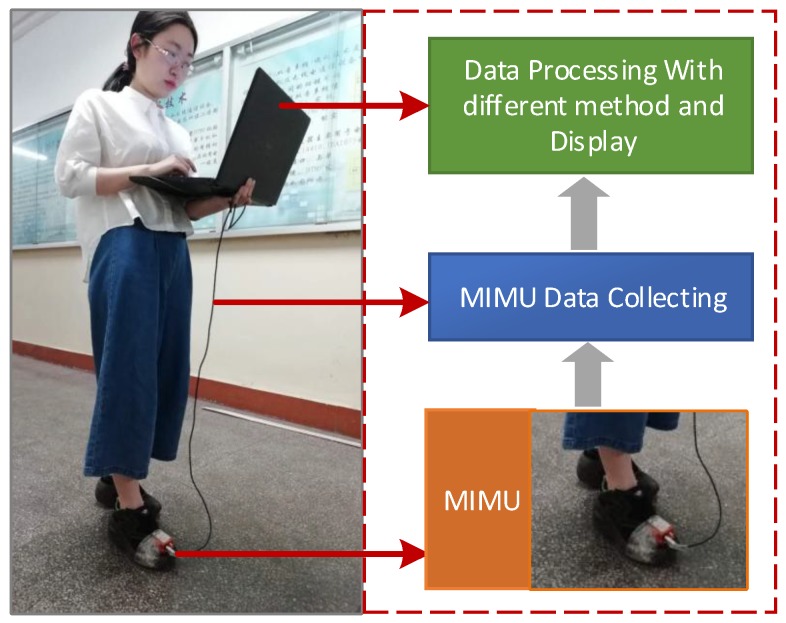
Pedestrian-positioning test structure.

The MTi-G710 MIMU integrates multiple sensors including accelerometers, gyroscopes, magnetometers, barometers, and GPS receivers. The MIMU is firmly connected with the foot in this experiment and the sampling frequency is 100 Hz. The device picture, installation position, and the corresponding software interface screenshot are shown in Figure 8. The MTi-G710 MIMU is used to collect the output of the accelerometers and gyroscopes and calculate the positioning results of the pedestrian in real time. The pedestrian-positioning test structure diagram is illustrated in Figure 9.

**Figure 10:**
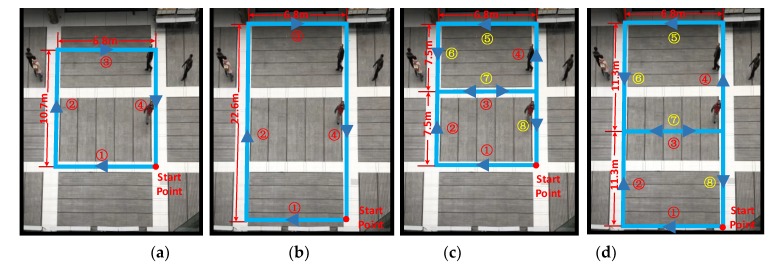
Pedestrian-positioning test route: (**a**) Route A; (**b**) Route B; (**a**) Route C; (**b**) Route D.

The four routes (routes A, B, C, and D) are selected as the walking trajectory of the pedestrian-positioning test, as illustrated in Figure 10. The starting point, trajectory and trajectory length are shown in Figure 10. The trajectory length is measured before the pedestrian-positioning test. There are two walking methods in four routes: uniform fast walking and slow-fast walking. Uniform fast walking is used to verify the applicability of the proposed pedestrian-positioning method, which is suitable for pedestrian fast movement; slow-fast walking is used to verify the stability of the proposed algorithm based on the correctness of the principle. The pedestrian-positioning test-related parameters for every test are illustrated in Table 2.

**Figure 11:**
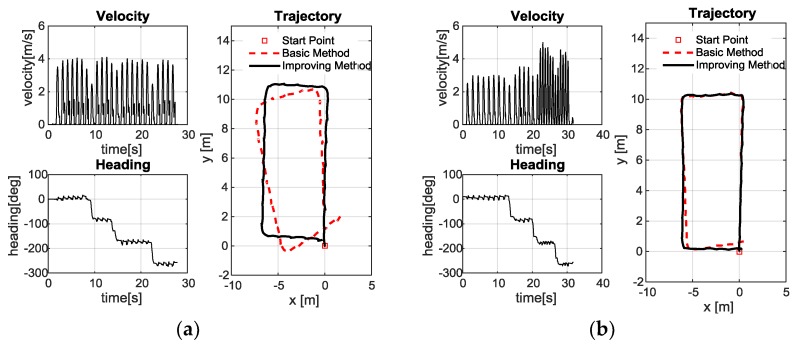

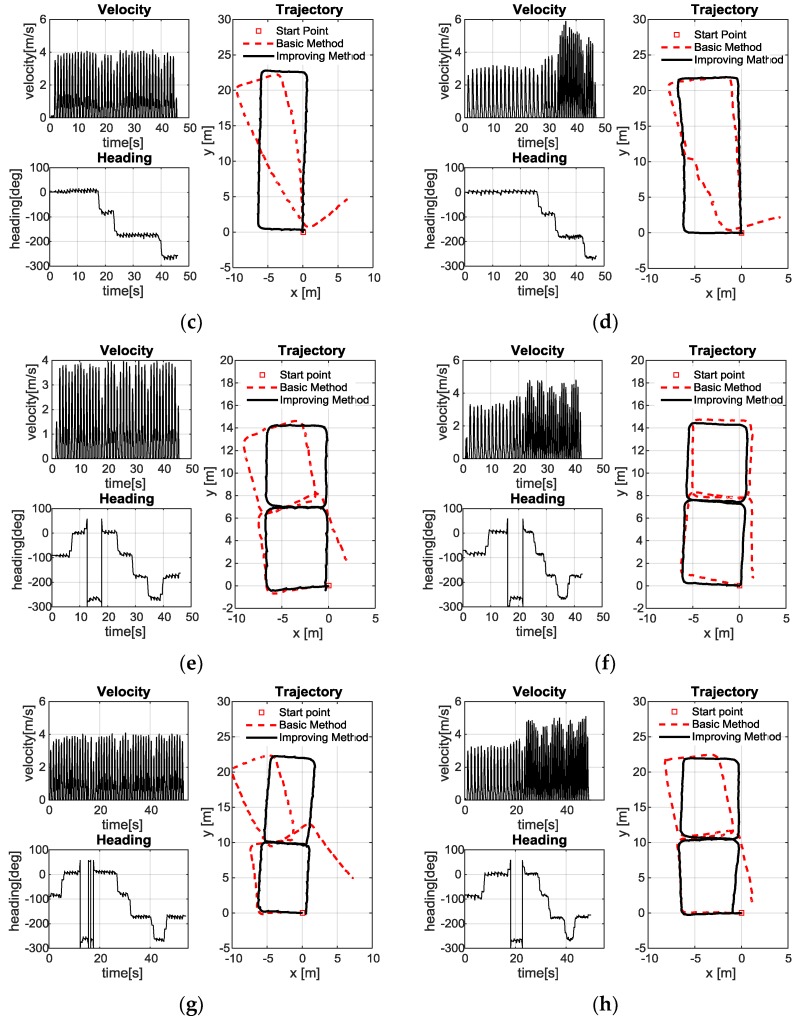
Pedestrian-positioning test results: (**a**) Test 1; (**b**) Test 2; (**c**) Test 3; (**d**) Test 4; (**e**) Test 5; (**f**) Test 6; (**g**) Test 7; (**h**) Test 8.

The pedestrian-positioning test result curves in Table 2 are illustrated in Figure 11. The changing curves of velocity and heading are introduced to describe the test state. Trajectory curves of pedestrian-positioning based on the traditional zero-velocity update and the proposed method are introduced to compare the principle correctness and improvement effect. The toe direction is introduced to describe the velocity direction.

Here, the basic method refers to the traditional zero-velocity update, which is the method without fuzzy logic or reverse solution. The improving method refers to the one using fuzzy logic and reverse solution. Since these experiments were conducted indoors where GPS signals cannot be properly received, the real track information cannot be obtained. Therefore, we can only provide an approximate hand drawing as shown in Figure 10. However, we have completed an outdoor pedestrian-positioning experiment where there is a real track using GPS signals, and we will show it in the following.

As shown in Figure 11, compared with the pedestrian positioning based on the traditional zero-velocity update, the proposed method effectively improves the pedestrian-positioning accuracy. Specifically, for fast walking (the maximum velocity is 4 m/s) in the same route (as shown in Figure 11a,c,e,g), a longer walking distance corresponds to a larger cumulative error of pedestrian positioning based on the traditional pedestrian-positioning method, whereas the positioning accuracy of the proposed forward and reverse calculation method based on the adaptive zero-velocity interval adjustment algorithm increases. For different velocities in the same route (as shown in Figure 11b,d,f,h), the improved effect of positioning is not obvious as the improved effect of the uniform fast walking test. The positioning accuracy is also significantly improved compared with the traditional positioning method because the velocity change motion is divided into two parts: slow walking (the maximum velocity is 3 m/s) and fast walking (the maximum velocity is 5 m/s). Slow walking does not pose a threat to the traditional positioning methods, i.e., when the maximum velocity is approximately 3 m/s, the traditional pedestrian-positioning method is less affected in terms of the positioning accuracy. The trajectories of the two methods in the first half of walking are consistent, as shown in Figure 11b,d,f,h. However, when the walking velocity increases (the maximum velocity is approximately 5 m/s), the applicability of the traditional pedestrian-positioning method deteriorates, and the positioning error rapidly increases. At this point, the proposed positioning method with forward and reverse calculation based on the adaptive zero-velocity interval adjustment algorithm has significantly improved the positioning accuracy. Thus, the proposed adaptive zero-velocity interval solution can effectively adjust the length of the interval adaptively and is suitable for different velocities.

**Figure 12:**
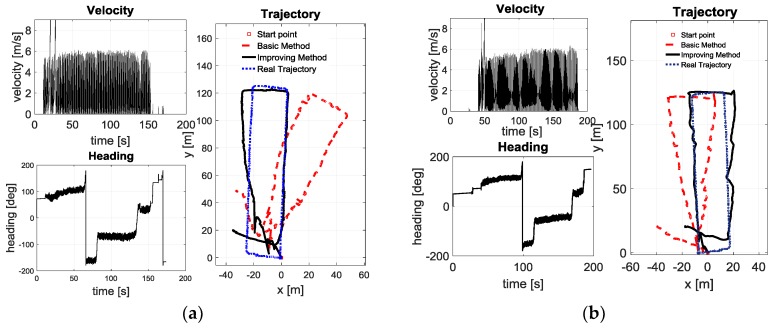
Long-distance running test results: (**a**) Test 1; (**b**) Test 2.

To further verify the effectiveness of the proposed method, long-distance running experiments were carried out outdoors. The two routes (routes 1 and 2) are selected as the running trajectory of the pedestrian-positioning test. The starting point, calculating trajectory and real trajectory are shown in Figure 12. The real trajectory and its length are obtained by GPS. The pedestrian-positioning test-related parameters for every test are illustrated in Table 3.

The pedestrian-positioning test result curves in Table 3 are illustrated in Figure 11. The changing curves of velocity and heading are used to describe the test state. Trajectory curves of pedestrian positioning based on the traditional zero-velocity update and the proposed method are used to compare the principle correctness and improvement effect.

As is shown in Figure 12, compared with the pedestrian-positioning based on the traditional zero-velocity update, the proposed method effectively improves the positioning accuracy when the pedestrian is running (the average velocity is above 6 m/s). The average deviation between the simulation trajectory calculated by the improving method and the real trajectory is no more than 3 m, and the maximum deviation is no more than 4 m. However, the average deviation between the simulation trajectory calculated by the basic method and the real trajectory is larger than 11 m, and the maximum deviation is larger than 45 m. In other words, the trajectory calculated by the improving method is smoother and closer to the real track. The main reason why the improving method performs better is that the adaptive zero-velocity interval adjustment method enables the system to detect the zero-velocity interval of the pedestrian’s foot more accurately, and the forward and reverse calculation makes the estimation of the pedestrian motion error information more accurate. Due to the long experimental path and the truth that both methods cannot estimate the heading error effectively, the simulation trajectories of the two groups of experiments have a relatively obvious deviation from the actual trajectory, but the improving method is still much better than the traditional zero-velocity update.

## 6. Conclusions

To solve the problem that the traditional pedestrian-positioning method based on foot-mounted MIMUs is unavailable for the fast movement of pedestrians, a forward and reverse calculation method based on the adaptive zero-velocity interval adjustment algorithm is proposed in this paper. According to the analysis of the effect of different walking speeds on the pedestrian-positioning accuracy, the zero-velocity interval detection during fast movement is inaccurate. To solve this problem, this paper presents an adaptive zero-velocity interval detection algorithm based on fuzzy logic reasoning, the concept of jerk is introduced to accurately determine the foot movement status of pedestrians, and the fuzzy logic reasoning method is introduced to self-adaptively adjust the data interval length of the zero-velocity judgment. Because the ground contact time of the foot is short during the fast movement of pedestrians, the accuracy of the zero-velocity update quickly decreases. To solve this problem, this paper presents a ZUPT method based on forward and reverse multiple solutions: with the forward and reverse processing of short-term data based on when the foot contacts the ground, a new set of data can be obtained to correct the positioning error. The MTi-G710, which is produced by the XSENS company, is used to complete the test. The experimental results verify the correctness and applicability of the proposed method.

## Figures and Tables

**Table 1:** Statistics of gait characteristics.

Number	Gait	Velocity	Step	Gait Cycle	Stance Phase	Data Length
1	Slow walking	2 km/h	70 cm	1.26 s	0.312 s	31
2	Normal walking	3 km/h	70 cm	0.84 s	0.208 s	20
3	Fast walking	5 km/h	70 cm	0.504 s	0.124 s	13
4	Slow running	7 km/h	90 cm	0.462 s	0.114 s	11
5	Normal running	10 km/h	90 cm	0.324 s	0.080 s	8
6	Fast running	15 km/h	90 cm	0.216 s	0.054 s	5

**Table 2:** Pedestrian-positioning test-related parameters.

Number	Route	Velocity	Route Length(m)	Movement Time (s)
1	A	Uniform velocity	35	27.8
2	A	Variable velocity	35	31.8
3	B	Uniform velocity	58.8	46.1
4	B	Variable velocity	58.8	47.4
5	C	Uniform velocity	57.2	46.2
6	C	Variable velocity	57.2	43.0
7	D	Uniform velocity	72.4	54.1
8	D	Variable velocity	72.4	49.8

**Table 3:** Long-distance running experiments-related parameters.

Number	Movement mode	Velocity	Route Length(m)	Movement Time (s)
1	Running	Uniform velocity	303	176
2	Running	Uniform velocity	305	198
